# Rosmarinic Acid Ameliorates Pulmonary Ischemia/Reperfusion Injury by Activating the PI3K/Akt Signaling Pathway

**DOI:** 10.3389/fphar.2022.860944

**Published:** 2022-05-11

**Authors:** Wenbin Luo, Yu Tao, Shengnan Chen, Hao Luo, Xiaoping Li, Shuang Qu, Ken Chen, Chunyu Zeng

**Affiliations:** ^1^ Department of Cardiology, Daping Hospital, Third Military Medical University, Chongqing, China; ^2^ Chongqing Key Laboratory for Hypertension Research, Chongqing Cardiovascular Clinical Research Center, Chongqing Institute of Cardiology, Chongqing, China; ^3^ State Key Laboratory of Trauma, Burns and Combined Injury, Daping Hospital, Army Medical University, Chongqing, China; ^4^ Cardiovascular Research Center of Chongqing College, Chinese Academy of Sciences, University of Chinese Academy of Sciences, Chongqing, China; ^5^ Department of Cardiology, Chongqing General Hospital, Chongqing, China; ^6^ Heart Center of Fujian Province, Union Hospital, Fujian Medical University, Fuzhou, China

**Keywords:** rosmarinic acid, pulmonary ischemia/reperfusion injury, oxidative stress, apoptosis, PI3K/AKT

## Abstract

Pulmonary ischemia/reperfusion (IR) injury is the leading cause of acute lung injury, which is mainly attributed to reactive oxygen species (ROS) induced cell injuries and apoptosis. Since rosmarinic acid (RA) has been identified as an antioxidant natural ester, this natural compound might protect against pulmonary IR injury. In this study, the mice were given RA daily (50, 75, or 100 mg/kg) by gavage for 7 days before the pulmonary IR injury. We found that hypoxemia, pulmonary edema, and serum inflammation cytokines were aggravated in pulmonary IR injury. RA pretreatment (75 and 100 mg/kg) effectively reversed these parameters, while 50 mg/kg RA pretreatment was less pronounced. Our data also indicated RA pretreatment mitigated the upregulation of pro-oxidant NADPH oxidases (NOX2 and NOX4) and the downregulation of anti-oxidant superoxide dismutases (SOD1 and SOD2) upon IR injury. *In vitro* studies showed RA preserved the viability of anoxia/reoxygenation (AR)-treated A549 cells (a human lung epithelial cell line), and the results showed the protective effect of RA started at 5 μM concentration, reached its maximum at 15 μM, and gradually decreased at 20–25 μM. Besides, RA pretreatment (15 μM) greatly reduced the lactate dehydrogenase release levels subjected to AR treatment. Moreover, the results of our research revealed that RA eliminated ROS production and reduced alveolar epithelial cell apoptosis through activating the phosphatidylinositol 3 kinase (PI3K)/protein kinase B (Akt) signaling pathway, which was supported by using wortmannin, because in the presence of wortmannin, the RA-mediated protection was blocked. Meanwhile, wortmannin also reversed the protective effects of RA in mice. Together, our results demonstrate the beneficial role of RA in pulmonary IR injury *via* PI3K/Akt-mediated anti-oxidation and anti-apoptosis, which could be a promising therapeutic intervention for pulmonary IR injury.

## Introduction

Acute lung injury is a common and severe disease with an incidence rate of 86.2 per 100,000 person-years and a mortality rate of 40% (Johnson and Matthay, 2010). Pulmonary ischemia/reperfusion (IR) injury is a pathological process observed in several clinical conditions, including infarction, thrombolytic therapy, and cardiopulmonary bypass (Eltzschig and Eckle, 2011), which is characterized by the injury of pulmonary alveolar epithelium and vascular endothelium due to reactive oxygen species (ROS) accumulation and inflammation during blood flow restoration (Oshima et al., 2016). The therapy of pulmonary IR damage mostly consists of nitric oxide inhalation, administration of prostaglandin 1, and complement inhibition as anti-inflammatory treatments, and their effectiveness has been proven in clinical studies (Aoe et al., 1994; Bacha et al., 1996; Zamora et al., 1999). In terms of scavenging oxidative stress, N-acetylcysteine was found to be effective in alleviating oxidative stress and improving the survival rate in transplant patients in a phase II clinical research (D’Amico et al., 2013). Meanwhile, other antioxidants, including methylene blue, melatonin, and creatine, have also been shown to attenuate pulmonary IR injury (Inci et al., 2002; Abreu et al., 2014; Almeida et al., 2016). However, those methods are not widely used due to their limited clinic effects or obvious side effects.

Rosmarinic acid (RA), as a common ester, exists in a variety of medicinal species within the plant genus Lamiaceae, such as basil, sage, rosemary, mint, and Perilla frutescens (Takano et al., 2004). Meanwhile, RA possesses the most potent antioxidant activity in hydroxycinnamic acids, and is commonly used to treat diseases such as peptic ulcers, arthritis, cataracts, cancer, rheumatoid arthritis, and bronchial asthma in clinics (Soobrattee et al., 2005; Petersen, 2013). Many physiological actions of RA, including anti-oxidation and anti-inflammation, have previously been described (Rašković et al., 2014). For instance, studies have shown that RA protects the heart, liver, and brain against IR injury through its antioxidant activity (Ramalho et al., 2014; Han et al., 2017; Zhang et al., 2017). It has been reported that RA could directly scavenge free radicals through the catechol hydroxyl group, and also play an indirect role by regulating the content of NADPH oxidases (NOX) and superoxide dismutases (SOD) (Imai et al., 2019; Liang et al., 2020). Thus, RA possesses the potential to protect the lung against IR injury through its antioxidant activity.

However, this possibility and the mechanism need further investigation. In the current study, we aimed to investigate the function and mechanism of RA in pulmonary IR injury. We provided evidence that RA could protect the lung against IR injury by reducing oxidative damage, inhibiting cell apoptosis and alleviating inflammation, which were regulated by the phosphatidylinositol 3 kinase (PI3K)/protein kinase B (Akt) signaling pathway. This study also provides a potential treatment or prevention drug for pulmonary IR injury.

## Materials and Methods

### Materials

RA was obtained from Aladdin, Shanghai, China. Schwartz Micro Serrefines were purchased from FineScienceTools, Germany. Kits for measurement of interleukin-1β (IL-1β), interleukin-6 (IL-6), lactate dehydrogenase (LDH), malondialdehyde (MDA), SOD, and NOX were from the Nanjing Jiancheng Bioengineering Institute, Nanjing, China. Wortmannin, AKT inhibitor VIII, hematoxylin and eosin (H&E) staining kit, Western and IP cell lysates, BCA protein assay kit, Dihydroethidium (DHE) kit, and Cell Counting Kit-8 (CCK-8) kit were obtained from Beyotime, Jiangsu, China. Protease inhibitor cocktails, and 4′, 6-diamino-2-phenyl-indole (DAPI) were obtained from Solarbio, Beijing, China. Nitro-cellulose membranes were purchased from BioTrace NT Nitrocellulose, PALL, United States. The *In Situ* Cell Death Detection Kit, POD was purchased from Roche, Mannheim, Germany. Rabbit anti-active caspase-3 antibody, rabbit anti-PI3K antibody, rabbit anti-p-PI3K antibody, rabbit anti-p-AKT antibody, and rabbit anti-BCL-2 antibody were from Cell Signaling Technology, Danvers, MA, United States. Rabbit anti-AKT antibody, rabbit anti-NOX2 antibody, rabbit anti-NOX4 antibody, rabbit anti-BAX antibody, and mouse anti-GAPDH antibody were obtained from ProteinTech, Wuhan, China. Rabbit anti-SOD1 antibody, and rabbit anti-SOD2 antibody were from Abcam, Cambridge, United Kingdom. Fluorescent-labeled goat anti-rabbit IgG, and fluorescent-labeled goat anti-mouse IgG were obtained from LI-COR Biotechnology, Lincoln, Nebraska, NE, United States.

### Animals

C57BL/6J male mice aged 10–12 weeks (weight 20–25 g) were obtained from the Experimental Animal Center of Daping Hospital (Chongqing, China). Mice were kept at room temperature (∼23°C) under a standard 12-h light/dark cycle.

Animal handling and surgical procedures were carried out in accordance with local institutional regulations and approved by the Experimental Animal Committee of Daping Hospital. All procedures were approved by the Research Council and Animal Care and the Use Committee of Daping Hospital, the Third Military Medical University (ethical approval number: AMUWEC20213011).

### Induction of Pulmonary Ischemia/Reperfusion Injury Model and Experimental Groups

The mouse model of pulmonary IR injury was carried out in accordance with the published protocol (Chen et al., 2017). Briefly, mice were anesthetized with an intraperitoneal injection of sodium pentobarbital (50 mg/kg) and placed on a heating pad to maintain body temperature. After tracheotomy, mice were ventilated with the Harvard ventilator (Hugo Sachs Elektronik) at a tidal ventilation of 7 ml/kg. The respiratory rate was set at around 100 breaths/min. After thoracotomy, lung ischemia was induced by clamping the left lung hilum using a Schwartz Micro Serrefine for 1 h, followed by 1 h of reperfusion. Subsequently, blood samples were immediately collected from the left ventricle for arterial blood gas analysis (Gem primer 3000, Instrumentation Laboratory). The oxygenation index (OI) was calculated by the ratio of partial pressure of oxygen (PaO_2_) to fraction of inspired oxygen (FiO_2_) (Xu et al., 2020; Wang et al., 2021). Meanwhile, other blood samples were placed at room temperature for 2 h. Then the blood samples were centrifuged for 3,000 rpm (30 min) to collect serum for enzyme-linked immunosorbent assay measurement of IL-1β and IL-6. Meanwhile, the left lungs were harvested and kept for further analysis.

Adult male mice were randomly subdivided into four groups: saline, RA 50 mg/kg, RA 75 mg/kg, RA 100 mg/kg, with 12 mice in each group. Then the mice were randomly divided into two parts in each group, one received sham operation, the other received pulmonary IR operation. According to the preliminary experiment, the optimal administration dose was 75 mg/kg *in vivo* (Joardar et al., 2019; Quan et al., 2021). Thereafter, the mice were randomly divided into four groups: Sham + Saline, Sham + RA 75 mg/kg, IR + Saline, and IR + RA 75 mg/kg, with six mice in each group (Lan et al., 2017). Furthermore, we used the PI3K inhibitor Wort (wortmannin, 1 mg/kg, intraperitoneally, 30 min before operation) in the pulmonary IR model (Yang et al., 2013; Liu et al., 2018b). Mice were divided into six groups: Sham + Saline, Sham + Wort, IR + Saline, IR + Wort, IR + RA 75 mg/kg, and IR + RA 75 mg/kg + Wort. In the Sham + RA 75 mg/kg, IR + RA 75 mg/kg, and IR + RA 75 mg/kg + Wort groups, mice were pretreated with RA (i.g.) for 7 days, and the same quantity of solvent was added to the sham or IR groups as RA 75 mg/kg pretreatment groups (Cao et al., 2019; Joardar et al., 2019).

### Cell Culture and Anoxia/Reoxygenation Treatment

A549, a lung epithelial cell line, was obtained from Procell Life Science & Technology in Wuhan and cultured in Dulbecco’s modified Eagle’s medium/high glucose with 10% FBS. Besides, RLE-6TN, a rat lung alveolar type II epithelial cell line, was cultured in Ham’s F-12 medium with 2 mM L-glutamine supplemented with bovine pituitary extract (0.01 mg/ml), insulin (0.005 mg/ml), insulin-like growth factor (2.5 ng/ml), transferrin (1.25 μg/ml), epidermal growth factor (2.5 ng/ml), and 10% FBS. The two cell lines were cultured in 1% v/v penicillin/streptomycin (Beyotime, Jiangsu, China) at 37°C.

After pretreatment with RA for 24 h (or saline as control), the cells were exposed to an anoxic chamber with 5% CO_2_ and 95% N_2_ at 37°C for 2 h followed by reoxygenation for up to 2 h (Jia et al., 2014; Chen et al., 2017). For inhibiting the PI3K/Akt signaling pathway, the PI3K inhibitor (wortmannin, 1 μM) or AKT inhibitor (AKT inhibitor VIII, 10 μM) was given to cells after replacing the medium with RA and incubated for 30 min before anoxia/reoxygenation (AR) (Xu et al., 2008; Liu et al., 2020). Cells were divided into three groups: Control + Saline, AR + Saline, and AR + RA to explore the effect of RA subjected to AR treatment, and then divided into four groups: Control + Saline, AR + Saline, AR + RA, and AR + RA + Wort to investigate the role of RA in PI3K/Akt-mediated protection.

### Wet/Dry Lung Weight Ratio Determination

The left lungs of different groups were weighed immediately after being removed to determine the wet weight, and then placed in an oven at 80°C for 24 h and re-weighed as dry weight to calculate the wet/dry lung weight ratio.

### Histological Analysis

The left lung samples were fixed in 4% paraformaldehyde for 1–2 days at 4°C and then dehydrated in ethanol. Thereafter, the samples were clarified in xylene and embedded in paraffin. Subsequently, samples were cut into 4-μm-thick sections and stained with an H&E staining kit, and the images were captured by the software viewpoint M8 Digital Scanning Microscopy System (PreciPoint, Freising, Germany). Subsequently, an experienced histologist from the Department of Virology of Daping Hospital scored on a four-point scale (Takıl et al., 2003) under blind conditions. Each section was randomly selected 10 microscope fields (×400) for histological analysis.

### Western Blot Analysis

Protein samples were extracted from lung tissues or A549 cells with Western and IP cell lysates containing protease inhibitor cocktails. The whole protein samples were collected and the concentrations were measured using a BCA protein assay kit. Tissue homogenates (50 mg of protein) were separated by 10% SDS-PAGE and transferred onto nitro-cellulose membranes. The blots were then washed with tris-buffered saline with Tween 20 (TBST), blocked with 5% milk powder in TBST buffer for 1 h, and incubated with the appropriate primary antibodies at appropriate dilutions. The blotted membranes were probed with the rabbit anti-active caspase-3 antibody (1:1,000), the rabbit anti-PI3K antibody (1:1,000), rabbit anti-p-PI3K antibody (1:1,000), rabbit anti-AKT antibody (1:1,000), rabbit anti-p-AKT antibody (1:1,000), rabbit anti-NOX2 antibody (1:500), rabbit anti-NOX4 antibody (1:500), rabbit anti-SOD1 antibody (1:1,000), rabbit anti-SOD2 antibody (1:1,000), rabbit anti-BAX antibody (1:1,000), rabbit anti-BCL-2 antibody (1:1,000), mouse anti-GAPDH antibody (1:10,000) at 4°C overnight. Then, the membranes were washed and primary antibodies were detected with fluorescent-labeled goat anti-rabbit IgG (1:15,000) or fluorescent-labeled goat anti-mouse IgG (1:15,000), and the bands were visualized by the Odyssey Western Blot Detection System (LI-COR Biotechnology, Lincoln, Nebraska, NE, United States). The images were analyzed using the Odyssey Application Software to obtain integrated intensities.

### TUNEL Assay

TUNEL assays were performed with the *In Situ* Cell Death Detection Kit, POD according to the manufacturer’s instructions. To visualize all nuclei, slides were incubated with DAPI. Finally, the Olympus DP80 digital camera (Olympus, Tokyo, Japan) was used to capture fluorescence images and the percentage of TUNEL-positive cells was calculated.

### Cell Viability Assay

Cell viability was assayed using CCK-8 (Beyotime, Jiangsu, China) according to the manufacturer’s protocol. Briefly, A549 cells were cultured in a 96-well plate, pretreated with RA (5, 10, 15, 20, 25 μM) or without RA for 24 h, and PBS was used as control. After normoxia or AR treatment, 10 μl CCK-8 solution/well was added and the cells were incubated for another 60 min at 37°C. The amount of formazan dye generated by cellular dehydrogenase activity was measured by absorbance at 450 nm with a microplate reader (ThermoFisher Scientific, Waltham, MA, United States).

### Lactate Dehydrogenase Detection

LDH is a stable cytoplasmic enzyme that can be quickly released into the cell culture medium when the plasma membrane is damaged, and is regarded as a marker of cell damage (Kumar et al., 2018). After RA (15 μM) pretreatment for 24 h, A549 cells were cultured in 96-well plates and then subjected to AR treatment. The LDH detection kit was used to measure the amount of LDH released from the cells, according to the manufacturer’s instructions (Dong et al., 2013).

### Dihydroethidium Staining

For detection of the superoxide anions, A549 cells in 24-well plates and tissue sections were incubated with 10 μM DHE at 37°C for 30 min. After being rinsed in PBS for three times, the fluorescence images were captured by an Olympus DP80 digital camera (Olympus, Tokyo, Japan) and analyzed using the Olympus Cellsens software.

### Detection of Malondialdehyde, Superoxide Dismutase and NADPH Oxidase

The levels of MDA, activities of SOD and NOX in lung tissues and A549 cells were detected by the colorimetric method using commercially available kits purchased from the Jiancheng Bioengineering Institute. All procedures were performed according to the manufacturers’ instructions.

### Statistical Analysis

Data were presented as mean ± standard deviation (SD) values. The data were analyzed using GraphPad PRISM software version 8.0 (GraphPad Software, Inc., San Diego, CA, United States) and SPSS 26.0 software (SPSS Inc., Chicago, IL, United States). Statistical significance was determined by the ANOVA test, followed by Sidak’s multiple comparisons test or Tukey’s multiple comparisons test for multi-group (>2) comparison. The statistic of the survival rate was analyzed by Kaplan-Meier curves and compared with the log-rank test followed by Bonferroni multiple comparison to determine the significance of difference. Differences were considered statistically significant when *p* < 0.05 as specified in the figure legends.

## Result

### Rosmarinic Acid Protects Against Pulmonary Ischemia/Reperfusion Injury

**FIGURE 1:**
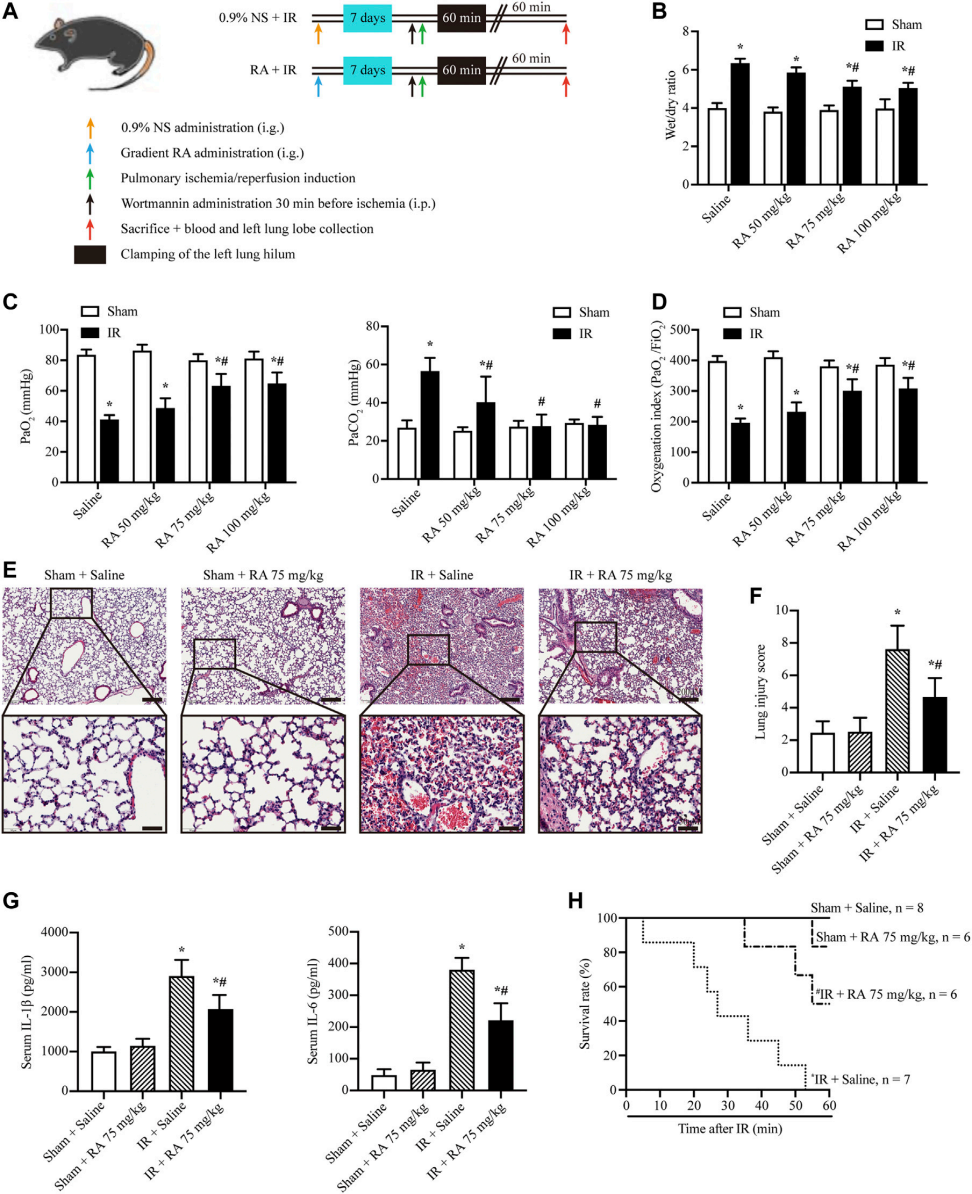
RA protects against pulmonary IR injury. **(A)** Schematic diagram of IR-induced lung injury in mice (RA: rosmarinic acid; IR: ischemia/reperfusion; NS: normal saline). **(B)** The wet/dry lung weight ratio of excised lungs from IR injury mice following 7 days of pretreatment with different dosages of RA was used to assess lung edema (*n* = 6; *p < 0.05 vs. Sham-Saline; ^**#**^ *p* < 0.05 vs. IR-Saline). **(C)** lung hypoxemia was evaluated as PaO_2_ and PaCO_2_ during IR injury (*n* = 6; *p < 0.05 vs. Sham-Saline; ^**#**^ *p* < 0.05 vs. IR-Saline). **(D)** OI (PaO_2_/FiO_2_) was calculated as a ratio to examine the respiratory dysfunction (*n* = 6; *p < 0.05 vs. Sham-Saline; ^**#**^ *p* < 0.05 vs. IR-Saline). **(E)** Histopathological changes in the IR-injured lung of mice pretreated with or without RA (scale bars, upper: 200 μm, lower: 50 μm). **(F)** The lung injury score of pulmonary tissues. Each group of staining was repeated at least 5 times, and 15 fields of view were selected for histopathological evaluation in each slice (*n* = 6; *p < 0.05 vs. Sham-Saline; ^**#**^ *p* < 0.05 vs. IR-Saline). **(G)** Serum concentrations of interleukin-1β (IL-1β) and interleukin-6 (IL-6) were measured using enzyme-linked immunosorbent assay (*n* = 6; *p < 0.05 vs. Sham-Saline; ^**#**^ *p* < 0.05 vs. IR-Saline). **(H)** The animal survival rate after pulmonary IR injury with RA pretreatment (*n* = 7 in IR + Saline group, *n* = 8 in Sham + Saline group, *n* = 6 in other groups; *p < 0.05 vs. Sham + Saline; ^**#**^ *p* < 0.05 vs. IR + Saline).

To explore the role of RA in pulmonary IR injury, the C57BL/6J mice were given different doses of RA (50, 75, or 100 mg/kg B.W/day) by gavage for 7 days before the pulmonary IR injury **(** Figure 1A **)**. We measured the wet/dry lung weight ratio as an evaluation of pulmonary edema. RA pretreatment with 50, 75, and 100 mg/kg significantly reduced pulmonary edema in mice subjected to IR injury, with optimal effectiveness at 75 and 100 mg/kg, while 50 mg/kg RA pretreatment was less pronounced (Figure 1B). Hypoxemia is another hallmark associated with lung function, which is indicated by PaO_2_, partial pressure of carbon dioxide (PaCO_2_) and OI (Jia et al., 2014, 53; Chen et al., 2017). The arterial blood gas analysis showed PaCO_2_ was increased while PaO_2_ and OI were decreased in mice subjected to pulmonary IR injury, while RA pretreatment (75 and 100 mg/kg) effectively prevented lung dysfunction (Figures 1C,D). Thus, we selected 75 mg/kg RA as the optimal therapeutic concentration in the following experiment. Moreover, the H&E staining and lung injury score revealed that the RA pretreatment reduced lung edema, blood cell exudation, and inflammatory cells infiltration in IR-induced lung injury (Figures 1E,F). The levels of proinflammatory cytokines IL-1β and IL-6 were elevated in serum derived from IR injury mice, and RA pretreatment reversed the cytokines elevation **(** Figure 1G **)**. Besides, we found that RA pretreatment significantly improved the survival of mice that were subjected to IR injury (Figure 1H). These results demonstrate that RA pretreatment has protective effects on lung function after IR injury by alleviating inflammation.

### Rosmarinic Acid Attenuates Ischemia/Reperfusion-Increased Oxidative Stress and Cell Apoptosis in Mice

**FIGURE 2:**
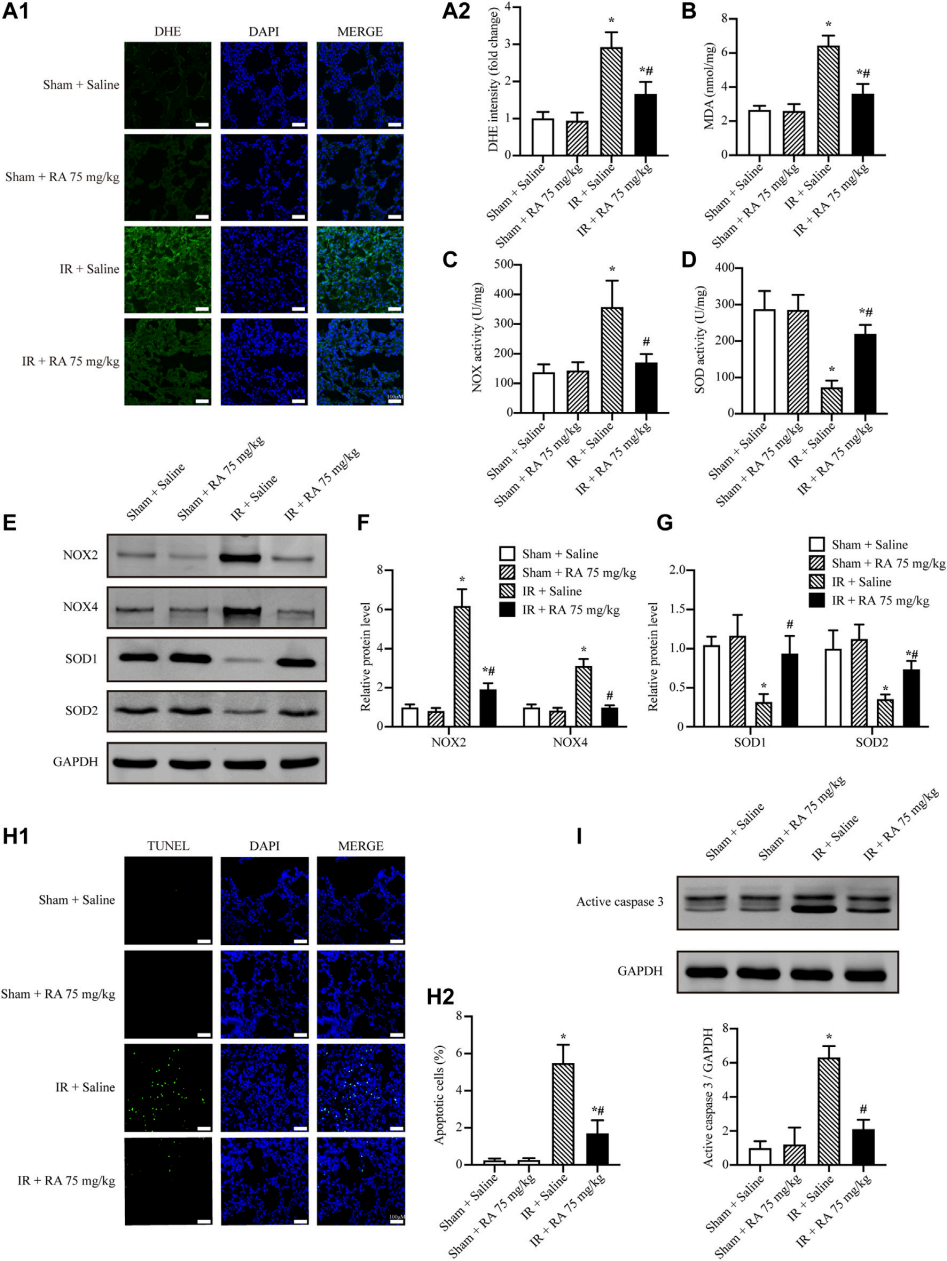
RA attenuates IR-increased oxidative stress and cell apoptosis in mice. **(A1,A2)** Representative micrographs of lung sections stained with DHE and semi-quantification analysis of fluorescence intensity indicated RA pretreatment reduced the ROS levels subjected to pulmonary IR injury (RA: rosmarinic acid; IR: ischemia/reperfusion; *n* = 6; *p < 0.05 vs. Sham + Saline; ^**#**^ *p* < 0.05 vs. IR + Saline; scale bars: 50 μm). **(B)** The expressions of MDA in lung tissues were detected (*n* = 6; *p < 0.05 vs. Sham + Saline; ^**#**^ *p* < 0.05 vs. IR + Saline). **(C,D)** The activities of NOX and SOD in lung tissues were detected (*n* = 6; *p < 0.05 vs. Sham + Saline; ^**#**^ *p* < 0.05 vs. IR + Saline). **(E–G)** NOX2, NOX4, SOD1 and SOD2 levels were detected using Western blots. The immunoblots were calculated by densitometric analysis using GAPDH as the internal reference (*n* = 3; *p < 0.05 vs. Sham + Saline; ^**#**^ *p* < 0.05 vs. IR + Saline). **(H1,H2)** TUNEL staining and statistical analysis showed RA pretreatment reduced cell apoptosis induced by IR injury (*n* = 6; *p < 0.05 vs. Sham + Saline; ^**#**^ *p* < 0.05 vs. IR + Saline; scale bar, 50 μm). **(I)** Representative blots and analysis results showed the active caspase 3 was elevated after IR injury and reversed by RA pretreatment (*n* = 3; *p < 0.05 vs. Sham + Saline; ^**#**^ *p* < 0.05 vs. IR + Saline).

Besides inflammation, we investigated the effect of RA pretreatment on IR-induced ROS levels and cell apoptosis in the lung tissues. Our results showed that RA pretreatment reduced IR-induced ROS and MDA elevations in the lungs (Figures 2A,B). Moreover, we revealed that pro-oxidant enzymes, NOX2 and NOX4, were upregulated while anti-oxidant enzymes, SOD1 and SOD2, were downregulated upon pulmonary IR injury. Noticeably, all these expressions of enzymes were reversed by RA pretreatment (Figures 2C–G). Our data also revealed that RA pretreatment reduced the increased number of apoptotic cells and the boosted activity of active caspase 3 upon IR injury (Figures 2H,I). Overall, these results suggest that RA pretreatment protects pulmonary function through anti-oxidation and anti-apoptosis.

### Rosmarinic Acid Reduces Oxidative Stress and Apoptosis Induced by Anoxia/Reoxygenation Injury in A549 Cells

**FIGURE 3:**
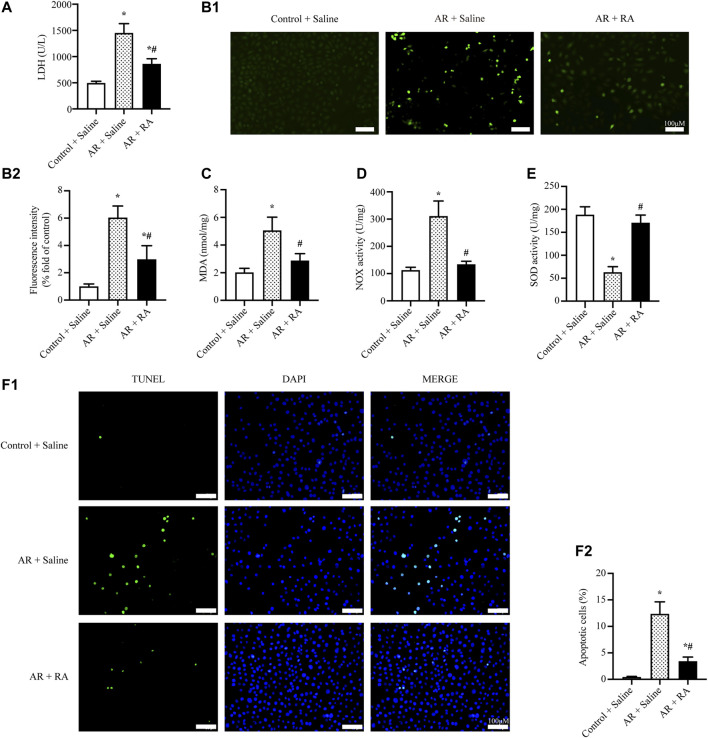
RA reduces oxidative stress and apoptosis induced by AR injury in A549 cells. **(A)** The release of LDH from A549 cells was reduced with RA (15 μM) pretreatment during AR injury (RA: rosmarinic acid; AR: anoxia/reoxygenation; *n* = 3; *p < 0.05 vs. Control + Saline; ^**#**^ *p* < 0.05 vs. AR + Saline). **(B1,B2)** Representative images and statistical analysis of DHE staining indicated that RA (15 μM) pretreatment reduced ROS content in AR-induced A549 cells (*n* = 3; *p < 0.05 vs. Control + Saline; ^**#**^ *p* < 0.05 vs. AR + Saline; scale bars: 100 μm). **(C–E)** The level of MDA and the activity of NOX were reduced while the activity of SOD was increased with RA (15 μM) pretreatment in AR-induced A549 cells (*n* = 3; *p < 0.05 vs. Control + Saline; ^**#**^ *p* < 0.05 vs. AR + Saline). **(F1,F2)** Representative pictures and the statistical results of the TUNEL assay illustrated that A549 cell apoptosis was alleviated with RA (15 μM) pretreatment when subjected to AR injury (*n* = 3; *p < 0.05 vs. Control + Saline; ^**#**^ *p* < 0.05 vs. AR + Saline; scale bars: 100 μm).

Alveolar epithelial cells are considered to be the principal target in pulmonary IR injury (Nicolls and Laubach, 2014). Thus, we further investigated the effect of RA on A549, a human lung epithelial cell line, which is used to investigate the function of alveolar epithelial cells (Giard et al., 1973). Here we determined the toxicity of RA in A549 cells at basal conditions, finding no obviously toxic effect of RA on cell viability after 24 h (Supplementary Figure S1A). Furthermore, based on the results of cell viability, we found RA pretreatment reduced AR-induced cell death, and the result showed the protective effect of RA started at 5 μM concentration, reached its maximum at 15 μM, and gradually decreased at 20–25 μM (Supplementary Figure S1B), which was consistent with previous research (Luan et al., 2013; Coelho et al., 2017). Thus, we selected 15 μM for subsequent experiments, finding that RA pretreatment (15 μM) greatly reduced cell death in A549 cells subjected to AR treatment measured by the LDH assay (Figure 3A). Meanwhile, it revealed the ability of RA to reduce AR-induced ROS levels, assessed by DHE staining and the MDA detection kit (Figures 3B,C). Similar results were obtained by LDH assay and DHE staining of RLE-6TN cells, a rat lung alveolar type II epithelial cell line (Supplementary Figures S2A,B). We also showed the increased SOD activity and decreased NOX activity were involved in the decreased ROS levels by RA in A549 cells (Figures 3D,E). Additionally, RA pretreatment reduced AR-induced cell apoptosis, as indicated by TUNEL staining (Figure 3F). Consistent with lung tissues, these data imply that RA protects pulmonary alveolar epithelial cells against oxidative damage and apoptosis through diminishing ROS accumulation.

### PI3K/Akt Signaling Pathway is Involved in the Protective Effect of Rosmarinic Acid on Oxidative Stress and Apoptosis Against Anoxia/Reoxygenation Injury

**FIGURE 4:**
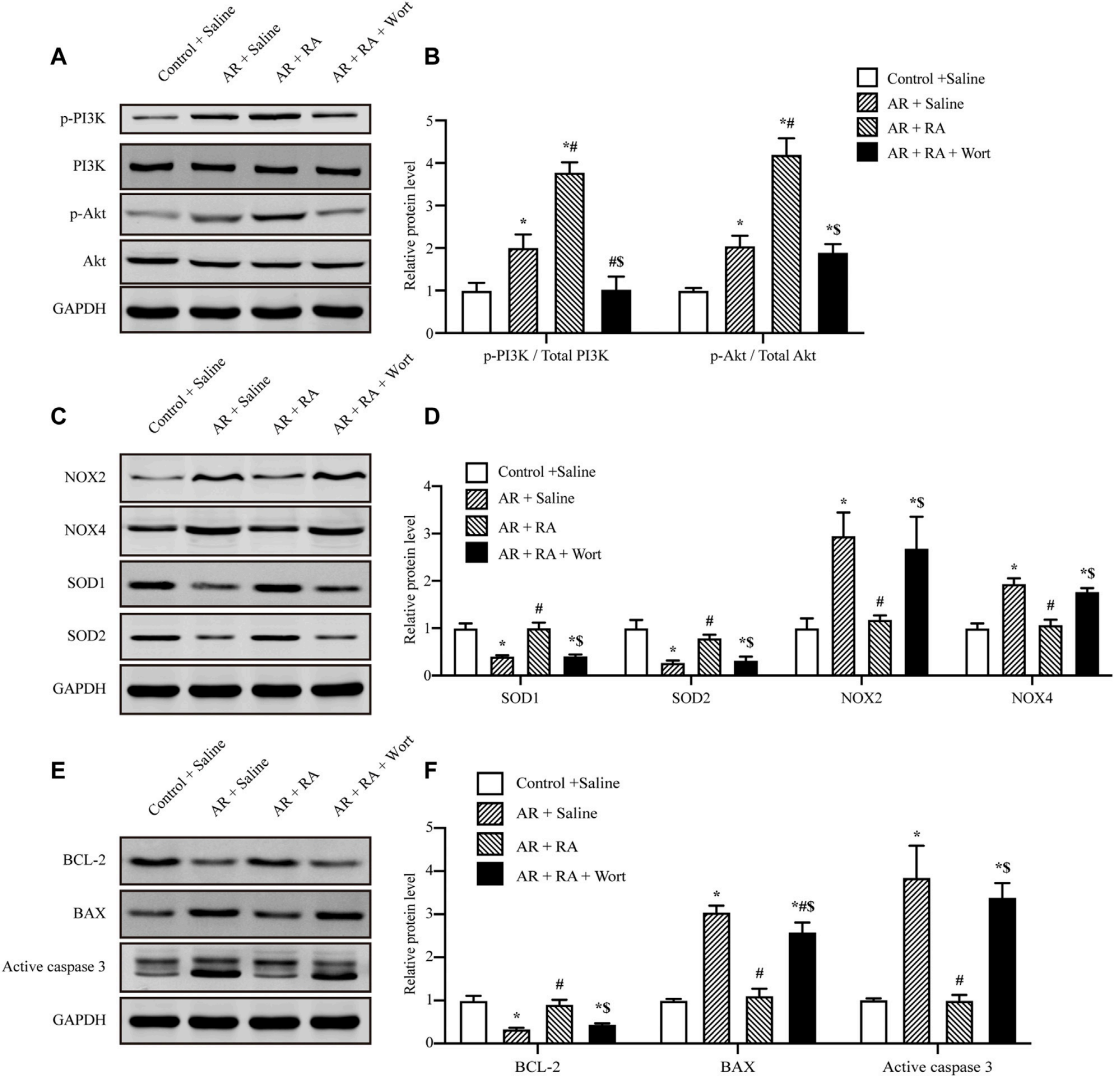
PI3K/Akt signaling pathway is involved in the protective effect of RA on oxidative stress and apoptosis against AR injury. **(A,B)** AR-induced PI3K and Akt phosphorylation increased with RA pretreatment (15 μM), but was blocked by wortmannin (1 μM). The immunoblots were calculated by densitometric analysis (RA: rosmarinic acid; AR: anoxia/reoxygenation; Wort: wortmannin; p-: phosphorylated; *n* = 3; *p < 0.05 vs. Control + Saline; ^**#**^ *p* < 0.05 vs. AR + Saline; ^$^ *p* < 0.05 vs. AR + RA). **(C,D)** Western blots were used to detect NOX2, NOX4, SOD1 and SOD2 levels. The immunoblots were calculated by densitometric analysis using GAPDH as the internal reference (*n* = 3; *p < 0.05 vs. Control + Saline; ^**#**^ *p* < 0.05 vs. AR + Saline; ^$^ *p* < 0.05 vs. AR + RA). **(E,F)** BCL-2, BAX and active caspase 3 levels were detected using Western blots. The immunoblots were calculated by densitometric analysis using GAPDH as the internal reference (*n* = 3; *p < 0.05 vs. Control + Saline; ^**#**^ *p* < 0.05 vs AR + Saline; ^$^ *p* < 0.05 vs. AR + RA).

RA could activate PI3K (Vlavcheski et al., 2017), and the PI3K/Akt pathway has been considered to play a vital role in the regulation of oxidative stress and apoptosis. Thus, we further administrated the PI3K inhibitor, wortmannin (1 μM) and Akt inhibitor VIII (10 μM) to explore whether RA protects AR injury through the PI3K/Akt signaling pathway, which was consistent with previous research (Xu et al., 2008; Liu et al., 2020). The cell viability assay indicated Wortmannin (1 μM) and Akt inhibitor VIII (10 μM) partially reversed the protective effects of RA on AR-induced A549 cell damage (Supplementary Figure S3). Furthermore, the phosphorylation levels of PI3K and Akt increased during AR injury, which were further increased by RA pretreatment, as compared with AR. However, in the presence of wortmannin, the increased phosphorylation of PI3K and Akt was inhibited (Figures 4A,B). Additionally, compared with AR injury, RA pretreatment downregulated the expression of pro-oxidative enzymes NOX2 and NOX4 and upregulated the expression of anti-oxidative enzymes SOD1 and SOD2, which were blocked by wortmannin (Figures 4C,D). These data indicate that RA might enhance Akt phosphorylation to regulate NOX and SOD activities. Similarly, RA pretreatment increased the anti-apoptotic protein B-cell lymphoma-2 (BCL-2) but decreased pro-apoptotic BCL2-Associated X (BAX) and, thus, inhibited the activity of active caspase 3, which were all blocked by wortmannin treatment (Figures 4E,F). Therefore, RA depends on activating the PI3K/Akt signaling pathway to inhibit oxidative stress and apoptosis in pulmonary alveolar epithelial cells.

**FIGURE 5:**
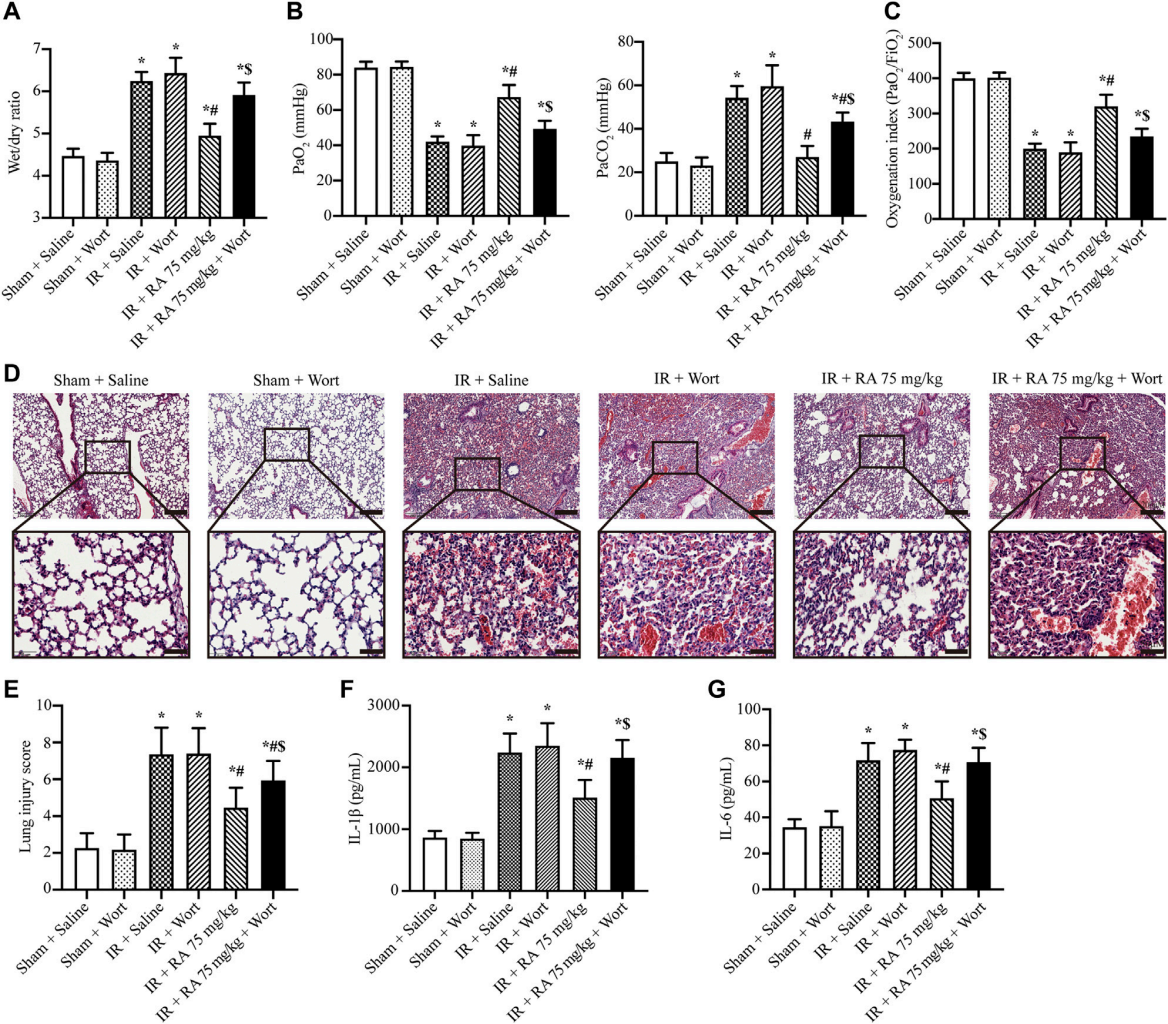
Effect of PI3K/Akt signaling pathway on the protection of pulmonary IR injury. **(A)** The wet/dry lung weight ratio of the excised lungs from mice demonstrated the effect of RA and wortmannin (1 mg/kg) administration on lung edema in IR mice (IR: ischemia/reperfusion; RA: rosmarinic acid; Wort: wortmannin; *n* = 6; *p < 0.05 vs. Sham + Saline; ^**#**^ *p* < 0.05 vs. IR + Saline; ^$^ *p* < 0.05 vs. IR + RA). **(B)** Lung hypoxemia was evaluated as plasma PaO_2_ and PaCO_2_ (*n* = 6; *p < 0.05 vs. Sham + Saline; ^**#**^ *p* < 0.05 vs. IR + Saline; ^$^ *p* < 0.05 vs. IR + RA). **(C)** OI (PaO_2_/FiO_2_) was calculated as a ratio to examine the respiratory dysfunction (*n* = 6; *p < 0.05 vs. Sham + Saline; ^**#**^ *p* < 0.05 vs. IR + Saline; ^$^ *p* < 0.05 vs. IR + RA). **(D)** Histopathological changes in IR-injured lungs of mice indicating RA pretreatment preserved the integrity of the lung structure, while wortmannin (1 mg/kg) reduced the protective effect (scale bars, upper: 200 μm, lower: 50 μm). **(E)** The lung injury score of pulmonary tissues. Each group of staining was repeated at least 5 times, and 15 fields of view were selected for histopathological evaluation in each slice (*n* = 6; *p < 0.05 vs. Sham + Saline; ^**#**^ *p* < 0.05 vs. IR + Saline; ^$^ *p* < 0.05 vs. IR + RA). The effects of RA on IL-1β **(F)**, IL-6 **(G)** levels in lung tissues during IR injury (*n* = 6; *p < 0.05 vs. Sham + Saline; ^**#**^ *p* < 0.05 vs. IR + Saline; ^$^ *p* < 0.05 vs. IR + RA).

These above data demonstrate that RA protects pulmonary IR injury *in vivo*, and the mechanism might be attributed to the PI3K/Akt signaling pathway to regulate ROS content *in vitro* (A549 cells). We further explored the role of the PI3K/Akt signaling pathway *in vivo*. Our results indicated that RA pretreatment alone reduced pulmonary edema, hypoxemia, and inflammation cytokines (IL-1β and IL-6) of the lung tissues following IR injury. The H&E staining indicated that the pretreatment of RA protected the structural integrity of lung tissue subjected to IR injury. In the presence of wortmannin alone (1 mg/kg), there was no significant effect on normal and IR-injured lung tissues, while the protective effect of RA was compromised in the presence of wortmannin and RA during IR injury (1 mg/kg) (Figures 5A–G).

## Discussion

Acute lung injury induced by pulmonary IR occurs in many clinical situations and becomes life-threatening in approximately 20% of patients (Sun et al., 2008). IR-induced pulmonary damage is characterized by nonspecific alveolar damage, pulmonary edema, and hypoxemia in clinics (Tang and Zhuang, 2019). At present, treatment is limited and narrowed to symptomatic and supportive care, which exhibits limited therapeutic effects. In recent years, natural compounds obtained from plants have received considerable attention. For instance, RA, possesses potent biological effects, has been shown to protect against IR injury in the heart and liver (Ramalho et al., 2014; Quan et al., 2021). However, the role of RA in pulmonary IR injury has not been reported yet. Thus, our study tried to explore the role of RA in the lung. Besides, more and more published reports have indicated the use of “drug preconditioning” or “drug pretreatment” to protect against limited operations. For instance, one study has implied a 7-day metformin pretreatment regimen significantly reduces postprocedural myocardial injury and improves 1-year clinical outcomes in patients undergoing percutaneous coronary intervention (Dirnagl et al., 2003; Li et al., 2014). In previous studies, pretreatment with folic acid for 1 week has been confirmed to ameliorate heart IR injury (Moens et al., 2008), while mice preconditioned with RA for 7 days show a prophylactic cardioprotective effect during myocardial IR injury (Quan et al., 2021). Thus, we pretreated mice with RA for 7 days and induced a pulmonary IR model. Our results found that 75 and 100 mg/kg RA pretreatment significantly alleviated hypoxemia and mitigated lung edema in pulmonary IR injury. Since the protective effects of the two concentrations on lung function and survival were approximately the same, we chose the relatively lower concentration of 75 mg/kg for further experiments. Lung function is largely dependent on its structural integrity, which is sensitive to inflammatory damage (Liu et al., 2018a). Therefore, we performed pathological staining and revealed that RA pretreatment reduced edema, inflammatory cells infiltration and blood cell exudation. Meanwhile, the levels of serum and lung tissue inflammatory factors were also reduced with RA pretreatment in IR injury. These were consistent with the findings that RA pretreatment inhibits the inflammatory cytokines through activating peroxisome proliferator-activated receptor-gamma (PPARγ) and down-regulating the nuclear factor kappa B (NF-κB)-mediated signaling pathway in the rat myocardial IR injury (Han et al., 2017). Besides, RA pretreatment improved the survival rate of mice subjected to IR injury. Thus, we conclude that RA improves lung tissue structure and function subjected to pulmonary IR injury by reducing inflammation damage.

During the pathological process of IR injury, massive ROS are produced as the initiating factor (Eltzschig and Eckle, 2011; Weissmann et al., 2012). For example, high levels of ROS directly oxidize cell membrane lipid to produce MDA and cause cell membrane structure injury (Kocaturk et al., 2020). In our study, we found that IR injury significantly increased ROS and MDA content in lung tissues and epithelial cells, but they were reduced by RA pretreatment. Previous studies have also shown RA protects against oxidative stress-induced liver and brain damage (Baranauskaite et al., 2020). Besides, lung tissue damage is the consequence not only of the direct effect of IR injury but also of ROS-mediated apoptosis (Valko et al., 2007; den Hengst et al., 2010). In our study, the decreased anti-apoptotic protein BCL-2, increased pro-apoptotic proteins BAX and caspase 3 activity in alveolar epithelial cells were inversed by RA pretreatment. These results were consistent with the previously reported study on the inhibition of apoptosis of RA (Lee et al., 2008). The above information indicates that RA protects lung alveolar epithelial cells and tissues, which are dependent on reducing IR-caused oxidative damage and apoptosis.

Furthermore, it’s widely accepted that the content of ROS is mainly regulated by the balance between ROS production and scavenging (Cui et al., 2018). The disrupted redox balance causes oxidative damage to lung tissue. NOX, especially NOX2 and NOX4, are the main source of cellular ROS production (Bedard and Krause, 2007; Katsuyama et al., 2011). On the other hand, cytoplasmic SOD1 and mitochondrial SOD2 are two of the most important endogenous ROS scavenging enzymes (Gerdprasert et al., 2019; Xie et al., 2020). In this study, RA pretreatment upregulated the expression and activities of antioxidant enzymes while downregulating the two pro-oxidant enzymes during IR injury, indicating that changes in the expression of oxidative kinases generally led to strong driving or damped alterations of enzyme activities and, ultimately, a low level of ROS. This result was similar to a previous study of RA on asthma (Liang et al., 2020). RA possesses the potential to eliminate ROS by chemical process in many diseases (Jang et al., 2018). However, the ROS elimination process needs further investigation in pulmonary IR injury. Summarily, RA protects pulmonary IR injury, at least in part, *via* increasing SOD1/2 activities and reducing NOX2/4 activities to reduce ROS content, subsequently alleviating oxidative damage and apoptosis.

The antioxidant effect of RA has been widely reported, and RA is even reported to be the most potent antioxidant among the hydroxycinnamic acids, which is the subclass of phenols (Soobrattee et al., 2005). However, the mechanism of RA regulation of NOX and SOD is unclear. Previous studies have reported that Akt activation inhibits the activity of NOX (Ying and Xiong, 2010) and upregulates the activity of SOD (Chen et al., 2019). In general, Akt acts as a downstream factor of PI3K, which is mediated by phosphoinositide-dependent kinase-1 (PDK-1) (Manning and Toker, 2017). Meanwhile, RA is a cinnamic acid derivative (Abd Aziz et al., 2021), and studies have shown that cinnamic acid could activate PI3K *via* binding to G-protein coupled receptors (GPCR) and Epidermal Growth Factor Receptor (EGFR) (Li et al., 2011; Kopp et al., 2014; Fruman et al., 2017). Therefore, we speculate that RA has a similar function in activating the PI3K/Akt signaling pathway, which is regarded as a critical event for survival and proliferation in various IR models (Dhanasekaran et al., 2008; Liu et al., 2020), while these effects could be abolished by coadministration with the PI3K specific inhibitor wortmannin, which binds to the p110 catalytic subunit of PI3K and irreversibly inhibits the enzyme (Ui et al., 1995; Ihara et al., 2020). Actually, previous studies have confirmed that the PI3K/Akt signaling pathway could be activated by RA (Vlavcheski et al., 2017). In our studies, we first assessed the expression and activities of NOX2/4 and SOD1/2 in lung tissues subjected to IR injury. Then we used alveolar epithelial cells to validate this phenomenon. Mechanistically, we found the PI3K/Akt signaling pathway was activated in AR treatment, which could be further activated by RA pretreatment with upregulated SODs activities and downregulated NOXs activities. Therefore, the ROS-induced apoptosis signal was also reduced. However, this activation effect of the PI3K/Akt signaling pathway and its downstream regulation of oxidative stress and apoptosis was removed in the presence of the PI3K inhibitor wortmannin *in vitro.* Moreover, in order to further clarify the clinical significance, mice were injected with wortmannin intraperitoneally 30 min before the IR injury. We evaluated a series of indicators, including pulmonary hypoxemia (PaO_2_, PaCO_2_, OI), lung edema, pathological staining, and levels of inflammatory factors in lung tissues. The analysis showed the protective effect of RA in pulmonary IR was abolished when PI3K was inhibited by wortmannin. These data indicate that RA could regulate NOXs and SODs activities through the PI3K/Akt signaling pathway.

Intriguingly, the changes in Akt phosphorylation in different animal models are variable. In our study, we detected that Akt phosphorylation was increased when subjected to IR injury. This phenomenon might be a compensatory response to stress. Previous studies found that short-term exposure to stress increases Akt activity, while long-term stress exposure inhibits the Akt signaling pathway (Chen et al., 2007; Tsai and Weissman, 2010). In our study, pulmonary IR injury was a relatively short-term injury (including 1 h for ischemia and 1 h for reperfusion), and phosphorylation of Akt was detected to upregulate during IR. The upregulation of Akt phosphorylation upon IR stress seems to be insufficient to inhibit NOX and compensate for SOD. When RA pretreatment, it further activates the PI3K/Akt signaling pathway, which ultimately inhibits NOXs, restores SODs, and protects lung IR.

The current study has several limitations. First, the potential mechanisms for RA-induced PI3K/AKT activation remain largely unknown. Although previous studies pointed out the cinnamic acid which derivates RA could activate PI3K by GPCR and EGFR, we still need further studies to reveal the underlying mechanisms. Second, since the cell viability analysis found RA improves cell survival in a dosage-dependent manner under AR insult, the pharmacokinetic parameters of RA are still unclear. And a further randomized control trial study could be performed to verify the therapeutical effect of RA in a further cohort.

## Conclusion

Our study found RA inhibits the activities of NOXs and increases the activities of SODs through activating the PI3K/Akt signaling pathway, thereby eliminating ROS, inhibiting oxidative damage and apoptosis, ultimately protecting lung structure and function in pulmonary IR injury. This study provides insight into a better explanation of the mechanism of RA, which may be used as a potential target for the treatment of clinical pulmonary IR injury.

## Data Availability

The original contributions presented in the study are included in the article/Supplementary Material, further inquiries can be directed to the corresponding authors.
