# Supplementary figures and images for: Rosmarinic Acid Ameliorates Pulmonary Ischemia/Reperfusion Injury by Activating the PI3K/Akt Signaling Pathway
Source: Front Pharmacol. 2022 May 11;13:860944. doi: 10.3389/fphar.2022.860944 (PMC9132383; doi:10.3389/fphar.2022.860944)

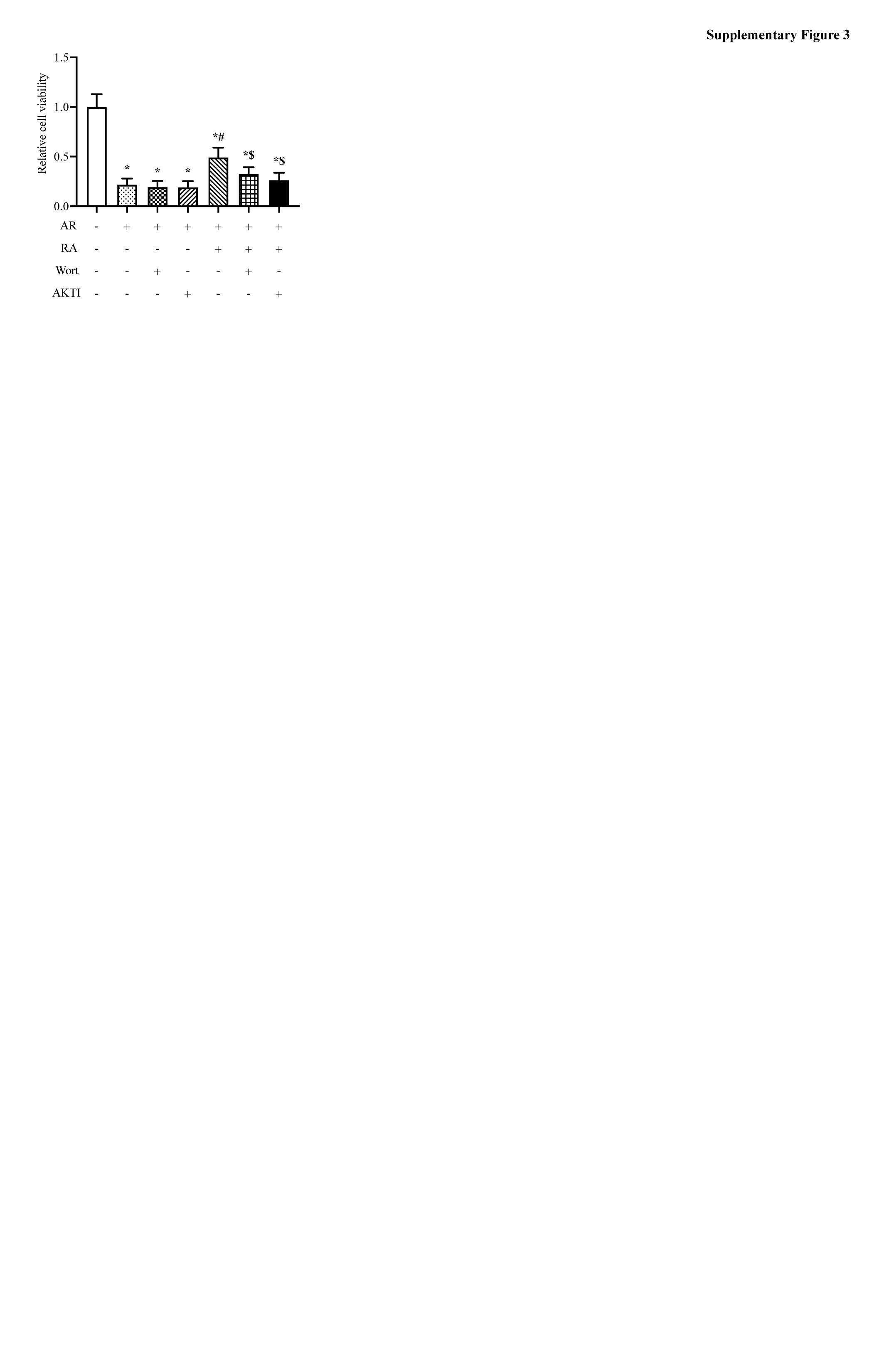

Supplement: Supplementary file 1 [file Image3.tif]

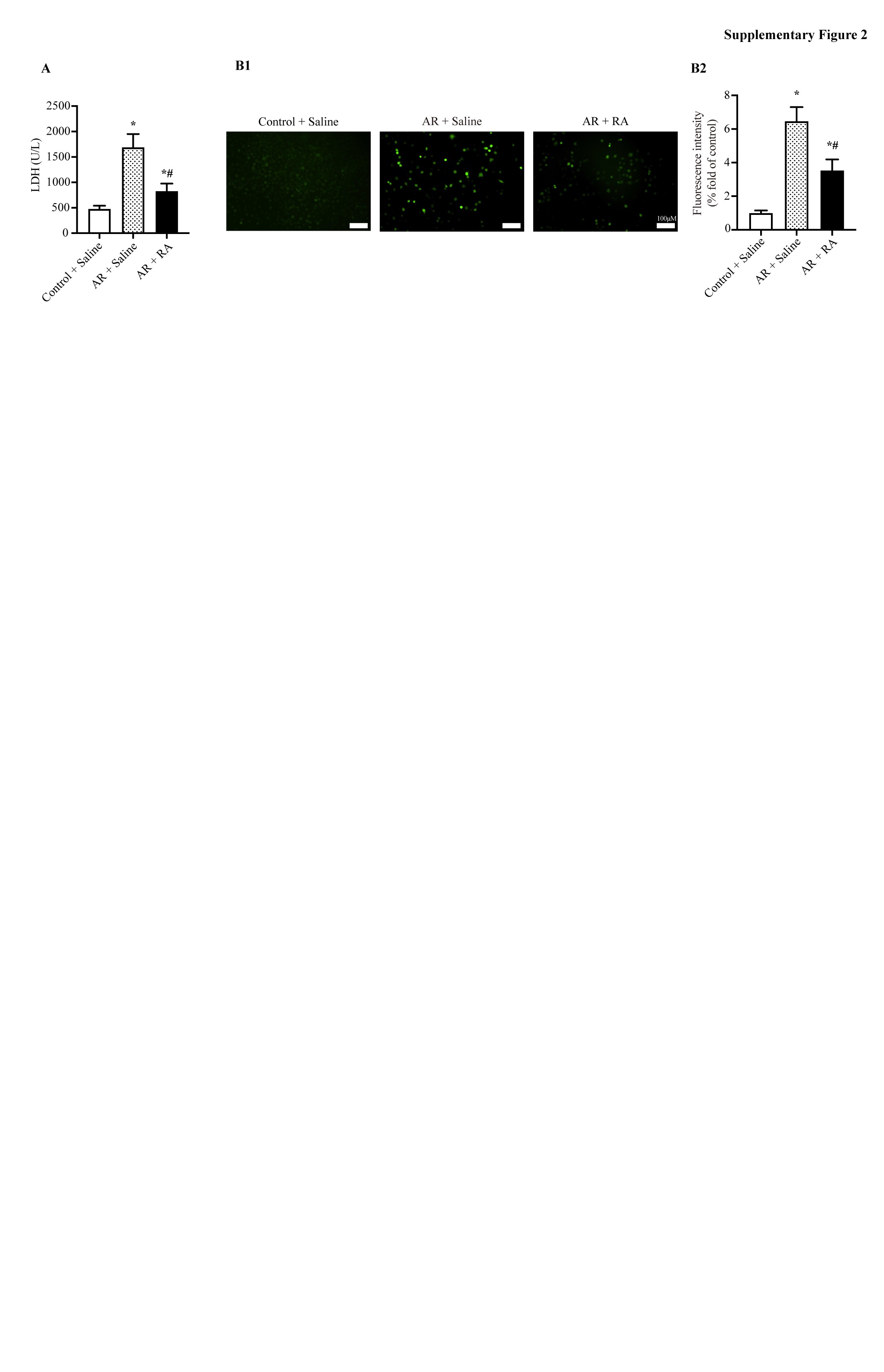

Supplement: Supplementary file 2 [file Image2.tif]

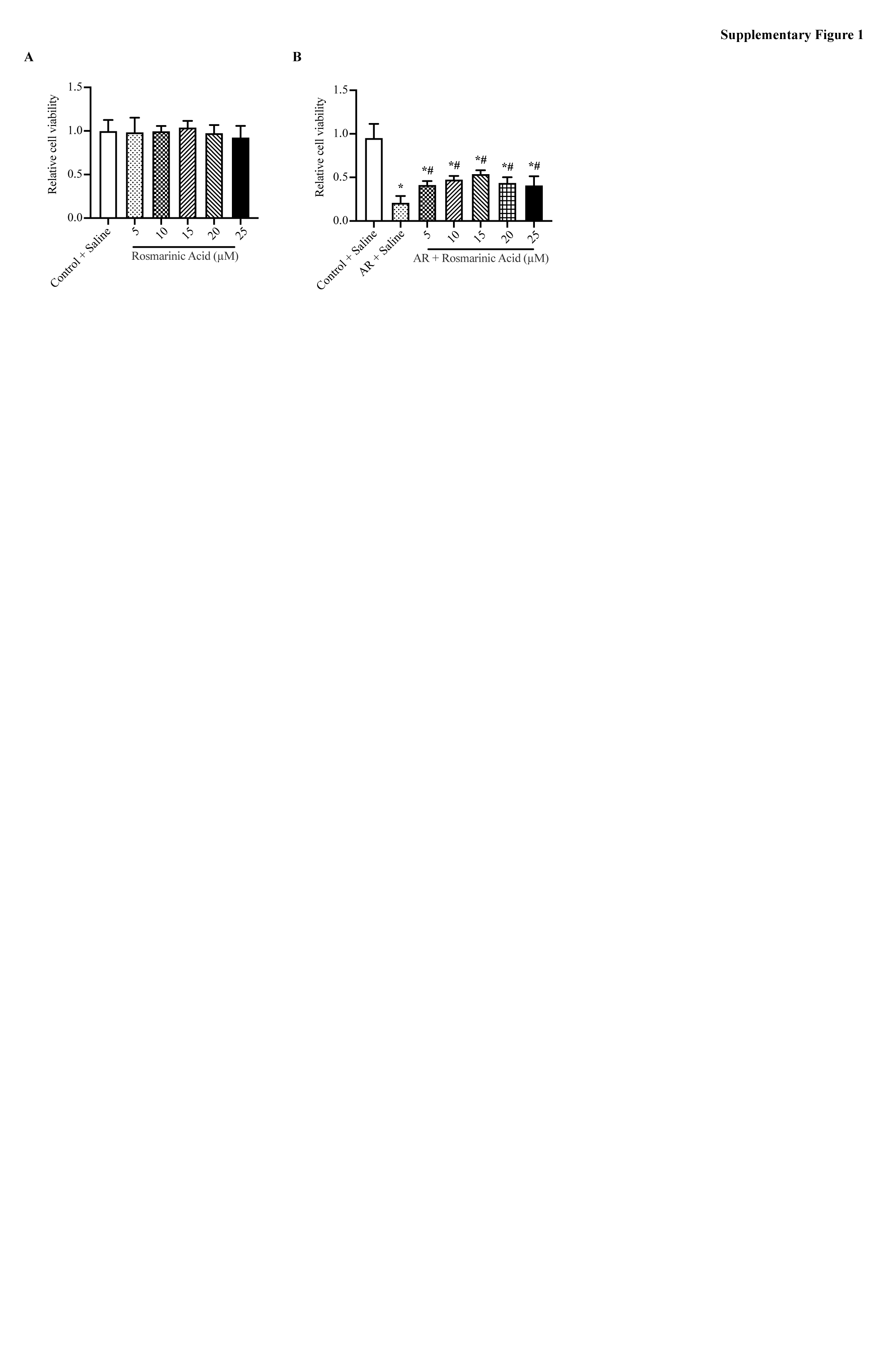

Supplement: Supplementary file 3 [file Image1.tif]
