# Supplementary material for: Rosmarinic Acid Ameliorates Pulmonary Ischemia/Reperfusion Injury by Activating the PI3K/Akt Signaling Pathway
Source: Front Pharmacol. 2022 May 11;13:860944. doi: 10.3389/fphar.2022.860944 (PMC9132383; doi:10.3389/fphar.2022.860944)
Supplement: Supplementary file 4 [file DataSheet1.docx]

Supplementary Material

# Supplementary Data

## Supplementary Figures


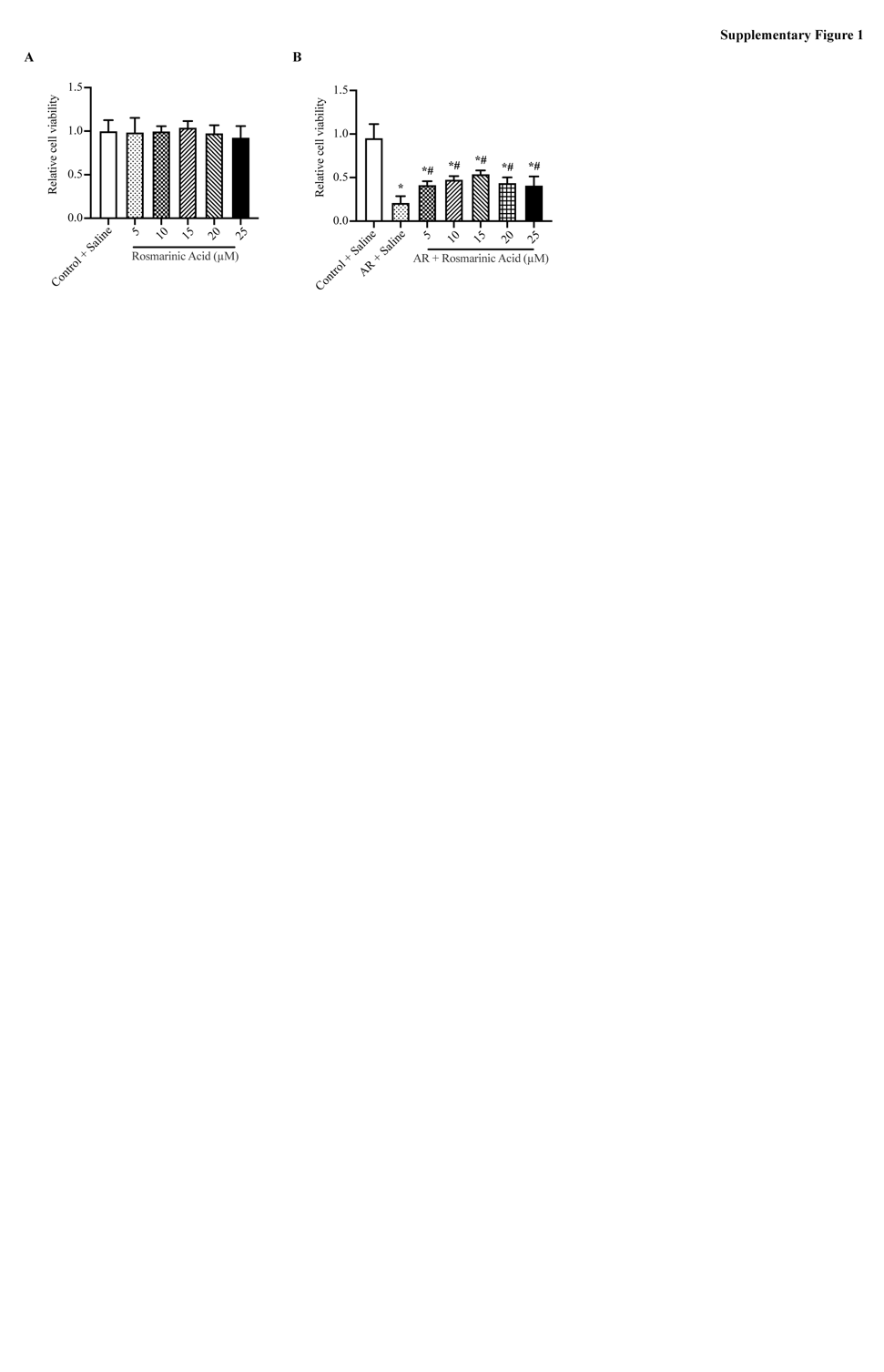


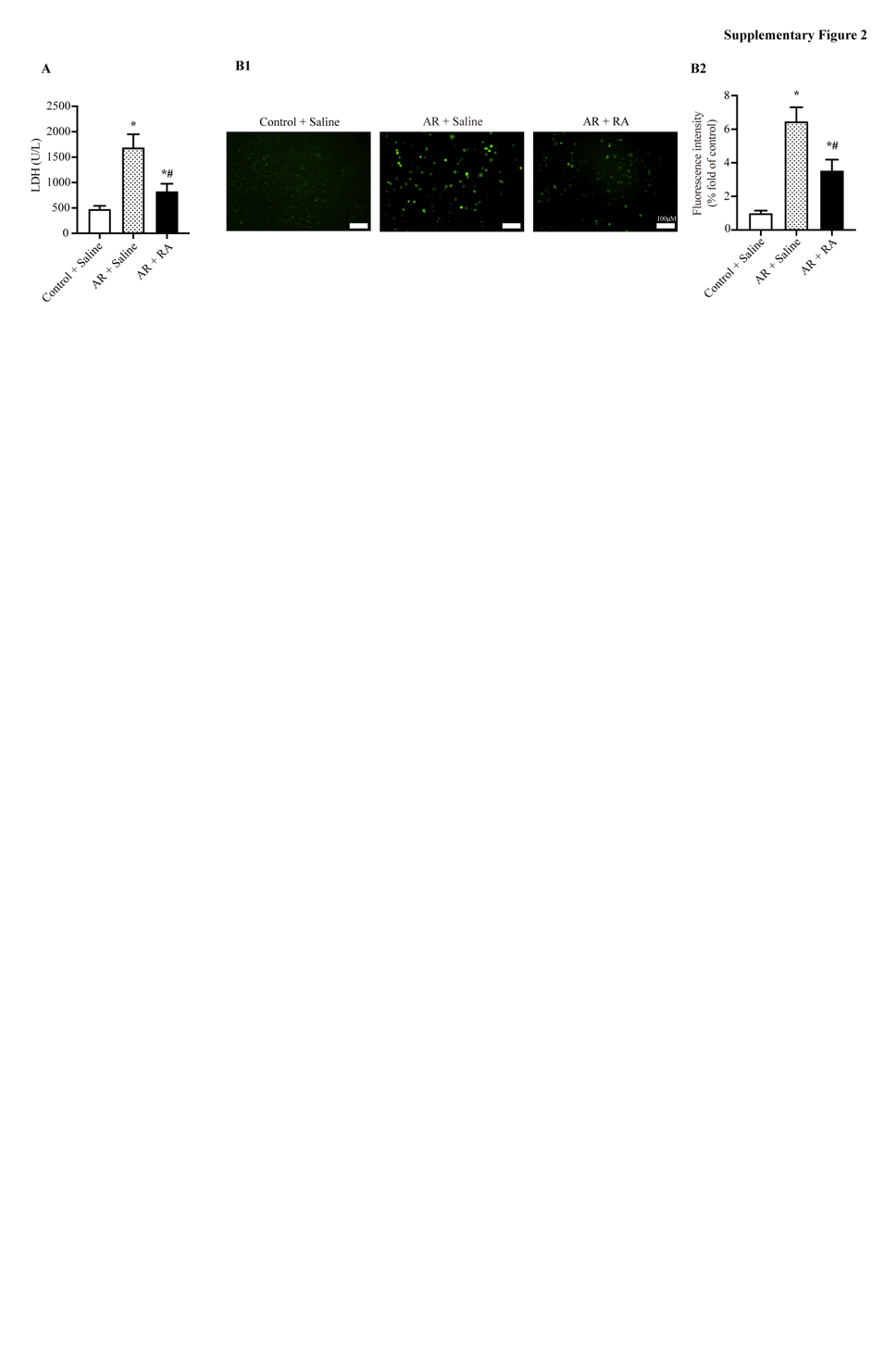
**Supplementary Figure 1** **| (A)** No obvious effect of RA (5-25 μΜ) on cell viability of A549 cells under normoxic conditions, indicated by the CCK-8 assay (RA: rosmarinic acid; n = 3; ******P* < 0.05 *vs.* Control group). **(B)** RA pretreatment preserved the viability of AR-induced A549 cells, as reflected by the CCK-8 assay (AR: anoxia/reoxygenation; n = 3; ******P* < 0.05 *vs.* Control group and **^#^***P* < 0.05 *vs.* AR group).


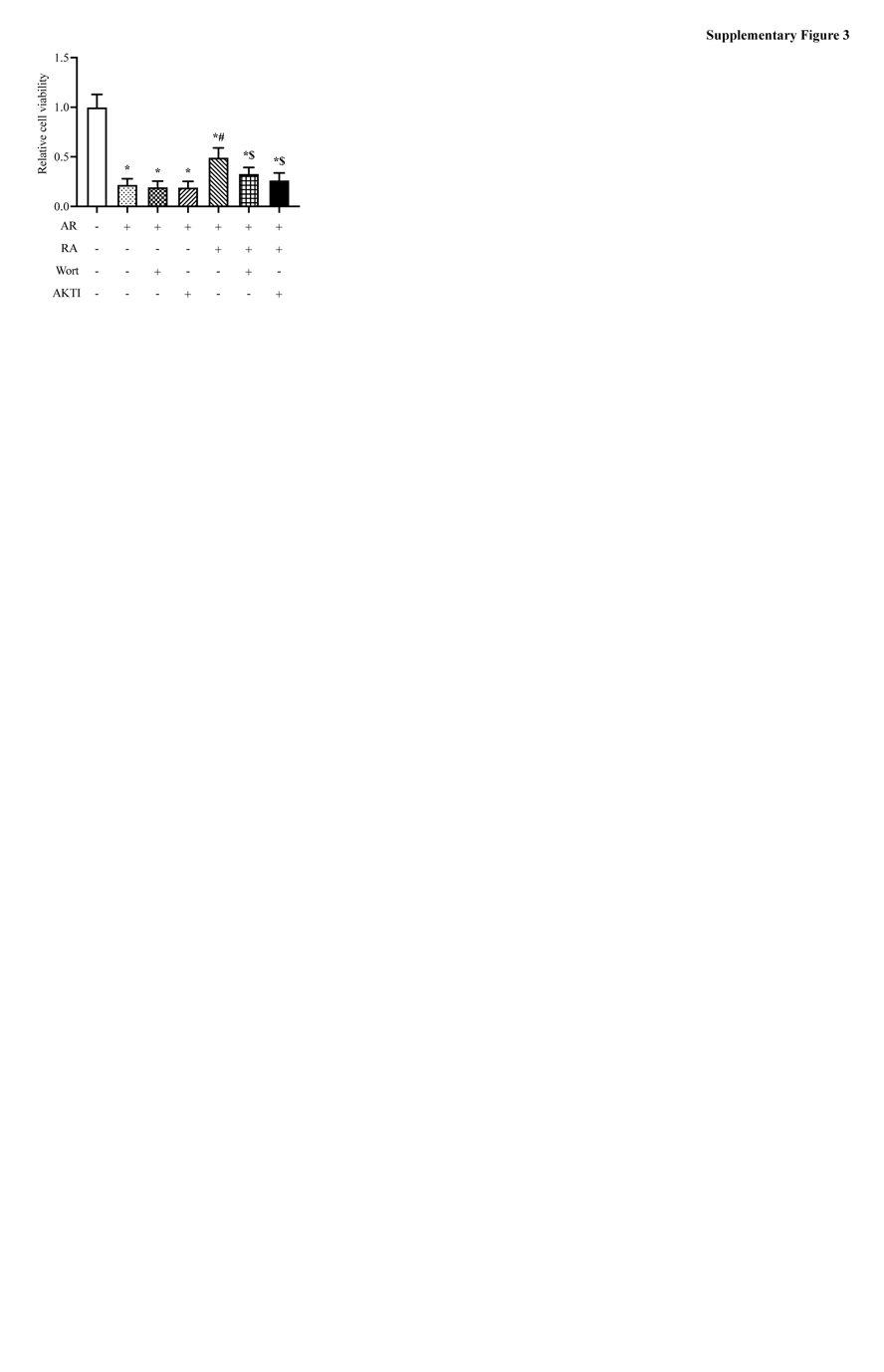
**Supplementary Figure 2 | (A)** The release of LDH from RLE-6TN cells was reduced with RA (15 μM) pretreatment during AR injury (RA: rosmarinic acid; AR: anoxia/reoxygenation; n = 3; ******P* < 0.05 *vs.* Control + Saline; **^#^***P* < 0.05 *vs.* AR + Saline). **(B1,B2)** Representative images and statistical analysis of DHE staining indicated that RA (15 μM) pretreatment reduced ROS content in AR-induced RLE-6TN cells (n = 3; ******P* < 0.05 *vs.* Control + Saline; **^#^***P* < 0.05 *vs.* AR + Saline; scale bars: 100 μm).

**Supplementary Figure 3 |** Wortmannin (1 μM) and Akt inhibitor VIII (10 μM) reduced the protective effect of RA on the viability of A549 cells exposed to AR injury using the CCK-8 assay (n = 6; ******P* < 0.05 *vs.* control + Saline group; **^#^***P* < 0.05 *vs.* the AR + Saline group; **^$^***P* < 0.05 *vs.* AR + RA group).
